# Global political responsibility for the conservation of albatrosses and large petrels

**DOI:** 10.1126/sciadv.abd7225

**Published:** 2021-03-03

**Authors:** Martin Beal, Maria P. Dias, Richard A. Phillips, Steffen Oppel, Carolina Hazin, Elizabeth J. Pearmain, Josh Adams, David J. Anderson, Michelle Antolos, Javier A. Arata, José Manuel Arcos, John P. Y. Arnould, Jill Awkerman, Elizabeth Bell, Mike Bell, Mark Carey, Ryan Carle, Thomas A. Clay, Jaimie Cleeland, Valentina Colodro, Melinda Conners, Marta Cruz-Flores, Richard Cuthbert, Karine Delord, Lorna Deppe, Ben J. Dilley, Herculano Dinis, Graeme Elliott, Fernanda De Felipe, Jonathan Felis, Manuela G. Forero, Amanda Freeman, Akira Fukuda, Jacob González-Solís, José Pedro Granadeiro, April Hedd, Peter Hodum, José Manuel Igual, Audrey Jaeger, Todd J. Landers, Matthieu Le Corre, Azwianewi Makhado, Benjamin Metzger, Teresa Militão, William A. Montevecchi, Virginia Morera-Pujol, Leia Navarro-Herrero, Deon Nel, David Nicholls, Daniel Oro, Ridha Ouni, Kiyoaki Ozaki, Flavio Quintana, Raül Ramos, Tim Reid, José Manuel Reyes-González, Christopher Robertson, Graham Robertson, Mohamed Salah Romdhane, Peter G. Ryan, Paul Sagar, Fumio Sato, Stefan Schoombie, R. Paul Scofield, Scott A. Shaffer, Nirmal Jivan Shah, Kim L. Stevens, Christopher Surman, Robert M. Suryan, Akinori Takahashi, Vikash Tatayah, Graeme Taylor, David R. Thompson, Leigh Torres, Kath Walker, Ross Wanless, Susan M. Waugh, Henri Weimerskirch, Takashi Yamamoto, Zuzana Zajkova, Laura Zango, Paulo Catry

**Affiliations:** 1 MARE—Marine and Environmental Sciences Centre, ISPA—Instituto Universitário, Lisboa, Portugal.; 2 BirdLife International, The David Attenborough Building, Pembroke Street, Cambridge CB2 3QZ, UK.; 3 British Antarctic Survey, Natural Environment Research Council, High Cross, Madingley Road, Cambridge CB3 0ET, UK.; 4 RSPB Centre for Conservation Science, Royal Society for the Protection of Birds, The David Attenborough Building, Pembroke Street, Cambridge CB2 3QZ, UK.; 5 U.S. Geological Survey, Western Ecological Research Center, Santa Cruz Field Station, 2885 Mission St, Santa Cruz, CA 95060, USA.; 6 Department of Biology, Wake Forest University, Winston Salem, NC 27109 USA.; 7 Department of Fisheries and Wildlife, Oregon State University, 104 Nash Hall, Corvallis, OR 97331, USA.; 8 Independent researcher, 204-100 Coe Hill Dr, Toronto, ON M6S 3E1, Canada.; 9 SEO/BirdLife, Marine Programme, C/Murcia 2-8, local 13, 08026 Barcelona, Spain.; 10 School of Life and Environmental Sciences, Deakin University, 221 Burwood Highway, Burwood, VIC 3125, Australia.; 11 Gulf Ecology Division, U.S. Environmental Protection Agency, Gulf Breeze, FL 32561, USA.; 12 Wildlife Management International Limited, P.O. Box 607, Blenheim 7240, New Zealand.; 13 Department of Environmental Management and Ecology, La Trobe University Albury–Wodonga Campus, Wodonga, VIC 3689, Australia.; 14 Oikonos Ecosystem Knowledge, Yerbas Buenas 498, Valparaíso, Chile.; 15 School of Environmental Sciences, University of Liverpool, Liverpool, UK.; 16 FitzPatrick Institute of African Ornithology, DST-NRF Centre of Excellence, University of Cape Town, Rondebosch 7701, South Africa.; 17 Department of Ocean Sciences, University of California Santa Cruz, Santa Cruz, CA 95064, USA.; 18 Institut de Recerca de la Biodiversitat (IRBio) and Department de Biologia Evolutiva, Ecologia i Ciències Ambientals (BEECA), Universitat de Barcelona, Av Diagonal 643, Barcelona 08028, Spain.; 19 World Land Trust, Blyth House, Bridge Street, Halesworth, Suffolk IP19 8AB, UK.; 20 Centre d’Etudes Biologiques de Chizé, CNRS La Rochelle Université, 79360 Villiers en Bois, France.; 21 The Hutton’s Shearwater Charitable Trust, 100 Watsons Road, Blenheim 7273, New Zealand.; 22 Associação Projecto Vitó, São Filipe, Fogo, Cabo Verde.; 23 Department of Conservation, Private Bag 5, Nelson, New Zealand.; 24 Department of Conservation Biology, Estación Biológica de Doñana, Consejo Superior de Investigaciones Científicas (EBD-CSIC), Avenida de Américo Vespucio, 26 Isla de la Cartuja 41092, Sevilla, Spain.; 25 Nature North, P.O. Box 1536, Atherton, QLD 4883, Australia.; 26 Shizuoka University, Johoku 3-5-1, Hamamatsu, Japan.; 27 CESAM, Departamento de Biologia Animal, Faculdade de Ciências Universidade de Lisboa, Rua Ernesto Vasconcelos, 1749-016 Lisboa, Portugal.; 28 Environment and Climate Change Canada, Wildlife Research Division, 6 Bruce Street, Mount Pearl, NL A1N 4T3, Canada.; 29 Biology Department, University of Puget Sound, 1500 N. Warner St., Tacoma, WA 98416, USA.; 30 Animal Demography and Ecology Unit, Institut Mediterrani d’Estudis Avançats (IMEDEA, CSIC-UIB), Miquel Marques 21, 07190 Esporles, Balears, Spain.; 31 UMR ENTROPIE (Université de La Réunion, IRD, CNRS, IFREMER, Université de Nouvelle-Calédonie) Université de La Réunion, Université de La Réunion, 15 Avenue René Cassin, CS92003, 97744 Saint Denis messag cedex 9, La Réunion, France.; 32 School of Biological Sciences, University of Auckland, Private Bag 92019, Auckland 1142, New Zealand.; 33 Auckland Museum, The Domain, Parnell, Auckland 1052, New Zealand.; 34 Auckland Council, Private Bag 92300, Victoria Street West, Auckland 1142, New Zealand.; 35 Oceans and Coasts, Department of Environment, Agriculture and Fisheries, Cape Town, South Africa.; 36 BirdLife Malta, 57/28 Marina Court, Abate Rigord Street, Ta’ Xbiex, XBX 1120, Malta.; 37 Psychology Department, Memorial University, St. John’s, NL A1B 3X9, Canada.; 38 WWF-Netherlands, Driebergseweg 10, 3708 JB Zeist, The Netherlands.; 39 Chisholm Institute, Bonbeach Campus, Breeze Street, Carmm, VIC 3197, Australia.; 40 Centre d’Estudis Avançats de Blanes CEAB (CSIC), Acces Cala Sant Francesc 14, 17300 Blanes, Spain.; 41 Tunisian Wildlife Conservation Society. Faculté des Sciences Mathématique, physique et biologiques de Tunis (FST), Campus Universitaire, El Manar, CP 2092 Tunis, Tunisia.; 42 Division of Avian Conservation, Yamashina Institute for Ornithology, 115 Konoyama, Abiko, Chiba 270-11, Japan.; 43 Instituto de Biología de Organismos Marinos (IBIOMAR), National Research Council of Argentina (CONICET), Bv. Almte Brown 2915, Puerto Madryn, Chubut, Argentina.; 44 Institute of Marine and Antarctic Studies, University of Tasmania, Commonwealth Science and Industrial Research Organization, CSIRO, Castray Esplanade, Hobart, Tasmania 7000, Australia.; 45 Wild Press, P.O. Box 12-397, Wellington, New Zealand.; 46 Independent researcher, 9 Roba Court, Kingston, Tasmania 7050, Australia.; 47 Université de Carthage Institut National Agronomique de Tunisie, 43 Avenue Charles Nicole, 1082 Tunis, Tunisie.; 48 National Institute of Water and Atmospheric Research Ltd., 10 Kyle Street, Riccarton, Christchurch 8011, New Zealand.; 49 Canterbury Museum, Rolleston Avenue, Christchurch 8053, New Zealand.; 50 Department of Biological Sciences, San Jose State University, One Washington Square, San Jose, CA 95192-0100, USA.; 51 Nature Seychelles Centre for Environment and Education, Sanctuary at Roche Caiman, Mahe, Seychelles.; 52 Halfmoon Biosciences, 45 Heather Road, Ocean Beach, WA 6333, Australia.; 53 Department of Fisheries and Wildlife, Oregon State University, Hatfield Marine Science Center, Newport, OR 97365, USA.; 54 National Institute of Polar Research, Tachikawa, Tokyo 190-8518, Japan.; 55 Mauritian Wildlife Foundation, Grannum Road, Vacoas, Mauritius.; 56 Department of Conservation, P.O. Box 10420, Wellington 6143, New Zealand.; 57 National Institute of Water and Atmospheric Research Ltd., 301 Evans Bay Parade, Hataitai, Wellington 6021, New Zealand.; 58 Department of Fisheries and Wildlife, Marine Mammal Institute, Oregon State University, Newport, OR 97365, USA.; 59 Institute of Marine Affairs and Resources Management, National Taiwan Ocean University, Keelung, Taiwan.; 60 Office of the Parliamentary Commissioner for the Environment, 2 The Terrace, Wellington 6011, New Zealand.; 61 Meiji Institute for Advanced Study of Mathematical Sciences, Nakano, Tokyo 164-8525, Japan.

## Abstract

Migratory marine species cross political borders and enter the high seas, where the lack of an effective global management framework for biodiversity leaves them vulnerable to threats. Here, we combine 10,108 tracks from 5775 individual birds at 87 sites with data on breeding population sizes to estimate the relative year-round importance of national jurisdictions and high seas areas for 39 species of albatrosses and large petrels. Populations from every country made extensive use of the high seas, indicating the stake each country has in the management of biodiversity in international waters. We quantified the links among national populations of these threatened seabirds and the regional fisheries management organizations (RFMOs) which regulate fishing in the high seas. This work makes explicit the relative responsibilities that each country and RFMO has for the management of shared biodiversity, providing invaluable information for the conservation and management of migratory species in the marine realm.

## Teaser

Highly-threatened seabirds connect many countries and the high seas and therefore depend on coordinated ocean governance.

## INTRODUCTION

The marine environment is characterized by high connectivity, which helps structure ecosystems and has important consequences for biodiversity conservation and human welfare (*1*, *2*). Distant waters are connected through the migrations of megafauna, including sea turtles, pelagic fish, marine mammals, and seabirds, as well as by ocean currents which drive the dispersal of fish larvae and other planktonic life forms (*3*–*5*). In addition to connecting natural systems, these processes also connect countries, which depend on shared biodiversity elements as resources for consumption, cultural identity, and associated ecosystem services but differ in their commitment to sustainable exploitation and conservation (*5*, *6*). During their seasonal movements, migratory marine animals visit areas under different legal and management regimes, including Exclusive Economic Zones (EEZs; up to 200 nautical miles from shore) where countries have resource rights, and areas beyond national jurisdiction (hereafter, the “high seas”) that are a global commons. Identifying the set of national jurisdictions and high seas areas visited by species across their seasonal cycles is vital, as strong management policy in one region may be nullified by unmitigated threats in another (*7*).

International cooperation and collaboration is key to the conservation of migratory species (*8*). On a global scale, countries can sign and actively engage in multilateral environmental agreements (MEAs), such as the Convention on Biological Diversity and the Convention on Migratory Species (CMS), and thereby contribute to international conservation by establishing common regulatory and guidance frameworks and setting global goals (*9*). Under CMS, there are a number of subsidiary agreements, such as the Agreement on the Conservation of Albatrosses and Petrels (ACAP), through which countries can share knowledge and promote policy measures relevant to specific elements of biodiversity (*10*). However, the degree to which these MEAs apply to the high seas is debated, as the obligations of signatory countries are restricted to areas and vessels under their jurisdiction (*11*). The primary global legal framework for maritime activities, the United Nations Convention on the Law of the Sea (UNCLOS), calls on countries to cooperate to preserve the marine environment both within national jurisdictions and in the high seas. However, explicit processes for enacting conservation and management measures for biodiversity in the high seas are lacking under UNCLOS, leaving a gap in legal responsibility (*12*).

The high seas have been documented as important habitat for an increasing number of migratory marine megafauna, yet, regardless, the footprint of human activities in the high seas continues to grow (*13*, *14*). Governance in the high seas is currently fragmented among institutional arrangements with sector-specific and/or regional mandates (*12*). The International Maritime Organization regulates shipping, for example, while regional fisheries management organizations (RFMOs) have mandates to regulate fishing. Fishing represents one of the largest industries operating in the high seas (*15*), and despite the formal commitment of RFMOs to ensure sustainable harvests and minimize broader ecosystem impacts, fisheries continue to represent a profound threat to high seas biodiversity (*16*).

To address the existing governance gap in the high seas, the United Nations have agreed to develop an international legally binding instrument under UNCLOS on the conservation and sustainable use of marine biological diversity of areas beyond national jurisdiction (aka “BBNJ treaty”). Core to the BBNJ treaty are provisions for protecting biodiversity through area-based management tools [e.g., marine protected areas (MPAs)] and the biodiversity-inclusive management of the wider ocean space, such as through the implementation of strategic environmental assessments and environmental impact assessments for planned activities (*11*, *17*). However, it remains unclear how existing management organizations and bodies will interact with the future processes adopted under the BBNJ treaty (*18*). For countries to be able to negotiate for, and ultimately implement, effective conservation and management measures under the new treaty and within the existing frameworks of MEAs and RFMOs, information is needed on the ways in which migratory species depend on and connect national jurisdictions and the high seas.

Tracking data from animals fitted with electronic devices have been used previously to estimate the proportion of time spent in different national jurisdictions and high seas areas and hence their relative importance to megafauna such as seabirds, sea turtles, tuna, sharks, and marine mammals (*13*, *19*–*21*). Tracking data can be integrated with population sizes to better quantify the importance of areas at sea, as sample-derived patterns can be contextualized with respect to the large-scale or regional distribution of each species (*22*, *23*). Population estimates are unreliable for many marine megafauna species because of their low detectability and wide distributions; yet, for seabirds, which assemble at predictable sites on land to breed, reasonably accurate estimations of population size can be used to weight estimates of space use at sea (*24*).

Albatrosses and large petrels have wide annual ranges spanning all major ocean basins. They are considered among the most threatened of all bird groups and are subject to several anthropogenic threats at sea, especially incidental mortality in fishing gear (termed “bycatch”) and competition with fisheries caused by overfishing, as well as pollution and climate-related changes to ecosystems (*25*, *26*). Using a unique tracking dataset assembled from across the global distributions of 39 species of albatrosses and large petrels, we estimate the importance of national jurisdictions and high seas areas across the annual cycle. First, we calculate species richness for each national jurisdiction (including the dependent territories under each sovereign country) and the high seas as a whole, distinguishing between breeding and visiting species (i.e., breeding in another country). Next, we estimate the amount of time, year-round, that each breeding population spends in each of these political areas, again discriminating between time spent in breeding-origin countries and other visited areas. Then, we attribute annual time spent in the high seas to the areas of competence of RFMOs. Last, we construct networks to reveal the geopolitical connections most important to the populations of each breeding-origin country and to the global community of albatrosses and large petrels. These detailed quantitative estimates of interconnectivity will inform countries on which jurisdictions their biodiversity depends and how to prioritize their engagement with the BBNJ treaty and other instruments and agreements.

## RESULTS

### Standalone importance of countries and the high seas

**Fig. 1:**
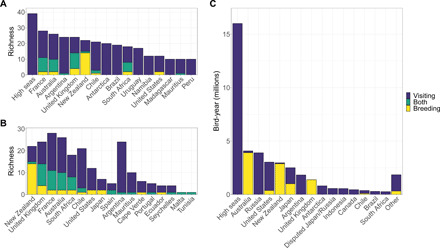
Standalone importance of countries and the high seas for albatrosses and large petrels. (**A** and **B**) Species richness within national jurisdictions and the high seas. For each political area, richness is divided into three categories: species that breed in the country (“Breeding”), species that visit the area but do not breed (“Visiting”), and species that both breed locally and visit from elsewhere (“Both”). (A) Top 15 areas in terms of total species richness. (B) Species richness of countries that host breeding populations, ordered by breeding richness. (**C**) Most important areas in terms of annual time spent, an index of abundance, by adult albatrosses and large petrels split into visiting and breeding bird components. Areas shown host more than 0.1% of annual time spent, with all others summed into the category “Other.” National jurisdictions refer to the aggregated EEZs (up to 200 nautical miles from shore) of each country, and high seas refers to all areas beyond national jurisdiction.

All 39 species of albatrosses and large petrels visited the high seas during their annual cycles, and except for the shy albatross *Thalassarche cauta*, all species visited the areas of jurisdiction of at least one country outside their breeding origin (Table 1, Fig. 1, and data S1). France had the highest total species richness (*n* = 28) and visiting-species richness (*n* = 26), while Argentina and Brazil had the highest number of species visiting that do not also breed in areas under their jurisdiction (*n* = 23 and *n* = 19, respectively; Fig. 1A). New Zealand had the highest number of breeding species (*n* = 15), and the United Kingdom had the highest richness of species that both breed in areas under their jurisdiction and visit from elsewhere (*n* = 10; Fig. 1B and data S1). In general, countries hosting a high diversity of breeding species also hosted a high number of visiting species, and countries with larger territories had higher richness (Fig. 1B and fig. S4). Brazil, Uruguay, Namibia, and Peru were each visited by ≥10 species although they have no breeding populations, and Antarctica (areas south of 60°S) was visited by 20 species (Fig. 1A and data file S1).

**Table 1:** Tracking data coverage of the global breeding population and annual cycle of 39 species of albatrosses and large petrels. “*N*_species_” is the estimated size (individuals) of the global breeding population. “*n*_sites_” is the number of breeding populations tracked per species. “*n*_birds_” is the number of unique birds tracked. “% Species pop.” is the percentage of the global breeding population made up by the sites tracked herein. “% Species year known” is the percentage of the annual cycle of the global breeding population estimated by our tracking data. “% Species year unknown” is the percentage of the annual cycle not estimated by our tracking data (i.e., months or populations without tracking data).

**Common name**	**Scientific name**	***N*_*s*pecies_**	***n*_sites_**	***n*_birds_**	**% Species pop.**	**% Species year** **known**	**% Species year** **unknown**
Amsterdam albatross	*Diomedea* *amsterdamensis*	90	1	74	100	100	0
Antipodean albatross	*Diomedea* *antipodensis*	14,864	2	211	100	100	0
Tristan albatross	*Diomedea* *dabbenena*	2,218	1	52	100	100	0
Wandering albatross	*Diomedea exulans*	18,568	5	977	100	100	0
Northern royal albatross	*Diomedea sanfordi*	10,270	2	75	100	92	8
Short-tailed albatross	*Phoebastria* *albatrus*	2,600	1	32	78	52	48
Laysan albatross	*Phoebastria* *immutabilis*	1,333,316	1	208	100	100	0
Waved albatross	*Phoebastria* *irrorata*	16,942	1	54	100	42	58
Black-footed albatross	*Phoebastria* *nigripes*	140,072	1	160	96	96	4
Sooty albatross	*Phoebetria fusca*	24,192	4	121	100	100	0
Light-mantled albatross	*Phoebetria* *palpebrata*	41,046	5	73	65	58	42
Buller’s albatross	*Thalassarche* *bulleri*	65,402	1	115	44	44	56
Indian yellow- nosed albatross	*Thalassarche* *carteri*	64,414	2	184	87	63	37
Shy albatross	*Thalassarche* *cauta*	29,368	1	143	100	83	17
Atlantic yellow- nosed albatross	*Thalassarche* *chlororhynchos*	67,300	1	45	100	92	8
Gray-headed albatross	*Thalassarche* *chrysostoma*	165,854	5	232	85	57	43
Chatham albatross	*Thalassarche* *eremita*	10,592	1	50	100	92	8
Campbell albatross	*Thalassarche* *impavida*	43,296	1	81	100	92	8
Black-browed albatross	*Thalassarche* *melanophris*	1,374,890	5	803	88	86	14
Salvin’s albatross	*Thalassarche* *salvini*	82,426	1	22	3	3	97
White-capped albatross	*Thalassarche* *steadi*	191,834	1	38	100	100	0
Buller’s shearwater	*Ardenna bulleri*	700,000	1	8	100	100	0
Flesh-footed shearwater	*Ardenna carneipes*	148,000	2	91	48	41	59
Pink-footed shearwater	*Ardenna creatopus*	67,040	1	102	100	92	8
Great shearwater	*Ardenna gravis*	5,000,000	1	72	100	100	0
Sooty shearwater	*Ardenna grisea*	20,000,000	2	54	46	45	55
Wedge-tailed shearwater	*Ardenna pacifica*	5,200,000	4	56	58	49	51
Short-tailed shearwater	*Ardenna* *tenuirostris*	23,000,000	1	16	78	72	28
Cory’s shearwater	*Calonectris* *borealis*	423,672	4	514	98	90	10
Scopoli’s shearwater	*Calonectris* *diomedea*	327,250	4	228	95	80	20
Cape Verde shearwater	*Calonectris* *edwardsii*	26,228	1	19	100	83	17
Streaked shearwater	*Calonectris* *leucomelas*	3,000,000	2	104	59	54	46
Southern giant petrel	*Macronectes* *giganteus*	95,406	5	243	25	19	81
Northern giant petrel	*Macronectes* *halli*	21,382	5	227	81	59	41
White-chinned petrel	*Procellaria* *aequinoctialis*	2,405,136	4	133	80	68	32
Gray petrel	*Procellaria cinerea*	151,132	4	61	98	89	11
Spectacled petrel	*Procellaria* *conspicillata*	28,800	1	8	100	50	50
Black petrel	*Procellaria* *parkinsoni*	3,000	1	61	100	92	8
Westland petrel	*Procellaria* *westlandica*	5,654	1	28	100	92	8
		64,302,254	87	5775	84.9	75.0	25.0

These species richness values were estimated directly from available tracking data; for several species, the sampled sites are not comprehensive of all breeding sites, and therefore, reported richness values are underestimates for certain countries. Breeding richness estimated from the tracking data was on average one species (mean, SD ±1.4) fewer than true breeding richness as calculated from the literature. Estimated richness was equal to true richness for 9 of the 22 countries that are known to have at least one breeding species of albatross or large petrel (data file S1). In most cases, missed breeding populations are relatively small in global terms, because our data included most of the global breeding population of each species (Table 1).

**Fig. 2:**
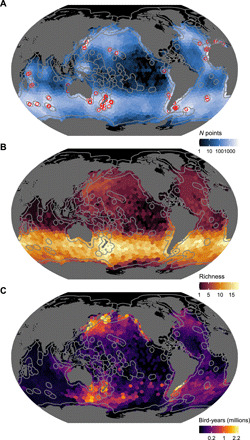
Tracking data and year-round distributions of albatrosses and large petrels. (**A**) Study sites (open red boxes) and the number of daily positions derived from tracked birds. (**B**) Species richness of adult birds in a breeding year. (**C**) Time spent during a breeding year. Cells indicate the total amount of time spent by the global breeding population of albatrosses and large petrels in a year. Gray lines at sea represent borders of national EEZs.

The high seas as a whole hosted the greatest amount of time spent by albatrosses and large petrels across the year, including 16 million of the total 41.3 million (39%) estimated bird-years (Fig. 1C). Among national jurisdictions, Australia had the highest annual time spent (i.e., by both visiting and breeding birds), with 4.0 million (9.6%) bird-years. Russia and the United States hosted the most time spent by visiting birds, representing an annual 3.9 million (9.4%) and 2.7 million (6.5%) bird-years, respectively (Fig. 1C and data S1). Australia and New Zealand each hosted the highest annual time spent by their own breeding populations, with a respective 3.9 million (9.4%) and 2.9 million (7.0%) bird-years (Fig. 1C and data S1). Static maps of annual species richness and time spent revealed contrasting patterns of albatross and large petrel diversity across the world (Fig. 2). The spatial pattern of time spent per month varied within a year, reflecting the dynamic seasonal distributions of albatrosses and large petrels (movie S1).

### Geopolitical connectivity

**Fig. 3:**
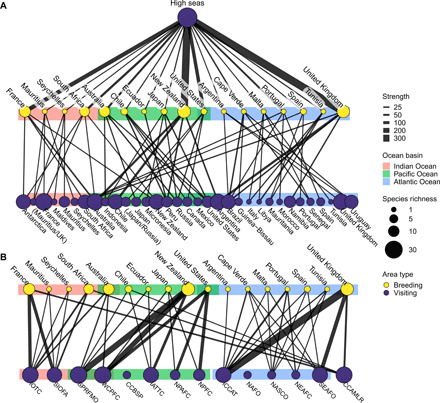
The most important geopolitical connections for albatrosses and large petrels. Connections are between breeding-origin countries (yellow dots) and visited national jurisdictions or high seas areas (purple dots), with dot size respectively representing the breeding and visiting richness in each and the link width signifying the strength of the connection. Connection strength is quantified as the percentage of annual time spent in the visited area, summed across the species making the connection. Annual time spent is calculated for each breeding population and weighted by the size of the population relative to global total of each species. (**A**) Top five connections between each breeding-origin country and the other areas visited throughout the annual cycle. (**B**) Top three connections between breeding-origin countries and the areas of competence of RFMOs in the high seas. Colored boxes represent the ocean basin in which most of the jurisdictional waters of each country or RFMO are located. RFMO abbreviations correspond to the following (left to right): Indian Ocean Tuna Commission, Southern Indian Ocean Fisheries Agreement, South Pacific Regional Fisheries Management Organisation, Western and Central Pacific Fisheries Commission, Convention on the Conservation and Management of Pollock Resources in the Central Bering Sea, Inter-American Tropical Tuna Commission, North Pacific Anadromous Fish Commission, North Pacific Fisheries Commission, International Commission for the Conservation of Atlantic Tunas, Northwest Atlantic Fisheries Organization, North Atlantic Salmon Conservation Organisation, North East Atlantic Fisheries Commission, South East Atlantic Fisheries Organisation, and Convention on the Conservation of Antarctic Living Marine Resources.

In terms of annual time spent, the high seas as a whole represent a “top connection” (i.e., one of the top five most visited areas) for the albatross and large petrel communities of all breeding-origin countries (Fig. 3A). At the global level, the breeding communities of the United Kingdom, France, and New Zealand have the strongest links to the high seas, indicated by the time spent there by the large breeding populations in these countries (i.e., proportions of all adult birds of each species), as well as the number of species making the connection. South Africa and Brazil were top connections for four separate breeding-origin countries (South Africa: France, Portugal, Spain, and United Kingdom; Brazil: Argentina, Cabo Verde, Portugal, and United Kingdom), and France, Peru, Mauritania, Russia, and the United States were among the top connections for populations breeding in three different countries (Fig. 3A and data S2A).

All high seas areas under the legal competence of RFMOs hosted a top connection (i.e., one of the top three most visited areas) with at least one breeding-origin country, with the exception of the smallest RFMOs (the North Atlantic Fisheries Organization and the Convention on the Conservation and Management of Pollock Resources in the Central Bering Sea; Fig. 3B). The International Commission for the Conservation of Atlantic Tunas (ICCAT), the Western and Central Pacific Fisheries Commission (WCPFC), and the South Pacific Regional Fisheries Management Organisation (SPRFMO) represented the most important high seas areas, in terms of the number of top connections with breeding-origin countries and the strength of connection (Fig. 3B and data S2B). The competence areas of ICCAT, WCPFC, and SPRFMO are among the largest in the world, and the size of RFMO areas was positively related to the total strength of their connections with breeding-origin countries (i.e., the sum of all connections per RFMO; Fig. 3B; *R*^2^ = 0.6, *P* < 0.001, df = 12; fig. S4).

## DISCUSSION

Migratory species cover immense distances on their seasonal movements, connecting numerous areas both within and beyond national jurisdiction and making their conservation a challenging task. By analyzing a comprehensive tracking dataset and accounting for the size of each breeding population relative to the global total, our study provides the first global estimation of the relative importance of political areas to a group of highly threatened migratory megafauna, the albatrosses and large petrels. We estimated the standalone importance of national jurisdictions and the connectivity between them, providing a potential road map for international collaboration. We also quantified the connections between breeding-origin countries and areas of legal competence of RFMOs in the high seas, providing a useful scoping tool for identifying which national populations of albatrosses and large petrels may be affected by management decisions in international fisheries. Our results showed that the high seas are important to albatrosses and large petrels from every country and in every ocean basin, indicating that effective management of international waters is of common, global interest.

By identifying the specific set of countries visited by albatrosses and large petrels originating in different countries, our findings provide support for existing bilateral and multilateral agreements and reveal additional connections of importance for these species. Our network confirms the connections, in terms of their albatross and petrel populations, among signatories of the CMS daughter agreement ACAP (which lists 29 of the 39 species studied herein) (*10*) and shows that bilateral agreements, such as the Japan-Australia Migratory Bird Agreement (http://www.austlii.edu.au/au/other/dfat/treaties/1981/6.html) or the New Zealand–Chile arrangement to protect seabirds (https://www.doc.govt.nz/news/media-releases/2018/chile-and-nz-arrangement-to-protect-seabirds/) including those affected by fisheries, have measurable importance. However, our results also reveal important gaps; for example, many birds from both Australia and Japan use Russian waters, yet there are no treaties or agreements between these countries that reflect this relationship. South Africa and Brazil host large numbers of birds from breeding populations across the Atlantic Ocean, and despite the engagement of these countries in CMS instruments, the most numerous species (i.e., *Calonectris* and *Ardenna* shearwaters) that contribute to these connections are not listed in CMS appendices and are therefore at risk of being omitted from international policy developments concerning migratory marine animals. To minimize gaps in protection, we suggest that any international conservation efforts for albatrosses and large petrels would benefit by considering the relative importance of countries for these species as identified herein.

Our maps of species richness and annual time spent reveal contrasting patterns in these two measures of biodiversity and identify hotspots both within and beyond national jurisdictions. Areas of high species richness were concentrated in the Southern Ocean, whereas areas of high annual time spent were generally distributed in the productive waters of continental shelf or upwelling regions, like the Patagonian Shelf in the South Atlantic Ocean and the Kuril Trench in the North Pacific Ocean. Notably, some areas of high annual time spent occurred in specific regions in the high seas, such as the Southern Ocean south of Tasmania, the northwest Pacific Ocean, and north-central Atlantic Ocean. The identified hotspots largely reflect the distributions of several species that are highly abundant trans-equatorial migrants, which number in the millions in the Atlantic (great shearwater *Ardenna gravis*) and Pacific Oceans (sooty *Ardenna grisea* and short-tailed shearwaters *Ardenna tenuirostris*). Our synthesis provides global snapshots of albatross and large petrel diversity, which have markedly seasonal distributions (see supplementary animation). The extensive and dynamic ranges of migratory marine megafauna, such as albatrosses and large petrels, indicate that static, area-based conservation measures alone, like MPAs, are not sufficient to address threats (*27*, *28*). Very large MPAs or those with dynamic management, with boundaries that shift across space and time, can be effective during the breeding season for certain species (*29*). However, during the nonbreeding season, when the movements of seabirds are less restricted, process-oriented and ecosystem-based management across the wider seascape is required (*27*). Regardless of the spatiotemporal scale of the approach, for any at-sea area-based protection to have positive outcomes for albatross and large petrel conservation, the implementation of effective fisheries measures is vital.

The high seas constitute the most important at-sea area for albatrosses and large petrels globally. Our estimates provide countries with tangible, quantitative indicators of the relative importance of the high seas for their respective communities of breeding seabirds, which are highly pertinent for countries involved in the ongoing discussions on the BBNJ treaty. The treaty objective is the conservation and sustainable use of high seas biodiversity; however, on the basis of the precondition of “not undermining” preexisting management organizations and bodies, fisheries have been excluded from treaty discussions (*18*). As the major, immediate at-sea threats to albatrosses and large petrels relate largely to fisheries (*25*), this exclusion may represent a missed opportunity to improve fisheries governance. Nevertheless, the BBNJ treaty could still influence RFMOs to the benefit of albatrosses and large petrels (*18*) in a number of ways—by formalizing a global process for establishing area-based management tools with appropriate regulations regarding bycatch and overfishing, by improving transparency and information sharing (particularly related to bycatch) via the establishment of an independent scientific committee, and by setting specific thresholds of impact that trigger the need for fisheries (or any other industry) to implement environmental impact assessments (*18*, *30*).

Our results also provide information to breeding-origin countries about the RFMO management areas in the high seas that are most important to each of their seabird populations. Despite being important because of their ocean-basin scale, the tuna RFMOs [ICCAT, WCPFC, Indian Ocean Tuna Commission (IOTC), and Inter-American Tropical Tuna Commission (IATTC)] are particularly relevant to albatross and large petrel conservation as industry practices have frequently been linked with overfishing and high bycatch rates (*16*, *31*). Fishing fleets operating in the high seas are under the jurisdiction of their flag states (*32*); therefore, despite not hosting albatrosses and large petrels within their national jurisdictions, some countries still affect them through poor fishing practices (*33*). The effectiveness of regulatory measures ultimately depends on the attitude of each country in terms of implementation and monitoring of compliance; however, as RFMOs operate on a consensus basis, it is the countries with a will to protect nontarget biodiversity that must push for effective regulation. Our results confirm that national measures for albatrosses and large petrels if coupled with coordinated, international efforts would successfully mitigate threats occurring across the ranges of these species. For example, while many species of conservation concern breeding on South African, French, United Kingdom, and Australian islands benefit from national action plans for reducing bycatch within national waters and fleets (*34*), birds are still at risk from longline effort in the high seas of the Indian Ocean, where they spend a considerable part of the year (Fig. 3B). Despite this, these countries have not advocated for the improvement of bycatch mitigation measures at the IOTC since 2012.

The tracking dataset used herein spanned the global ranges and annual cycles for most of our focal group, the albatrosses and large petrels, providing a uniquely representative picture of their global distribution (mean coverage of 85 and 75%, respectively; Table 1). Nevertheless, gaps remain in terms of untracked populations and unsampled months of the year. Using knowledge of known breeding sites and population sizes, we quantified these sampling limitations (Table 1) and thereby identified gaps at the species level where future work may be focused (*35*, *36*). Tracking and population data were only analyzed for breeding adults, and therefore, our results pertain to the global breeding populations of each species in a given year. A substantial proportion of the global population is made up of juveniles, immatures, and deferring breeders (particularly for biennial species), which may have different annual distributions and hence geopolitical connectivity (*37*, *38*). These are important gaps to fill in terms of tracking and monitoring effort; however, as these data accumulate, syntheses across ranges, taxonomic groups, and life stages can become standard practice (*24*).

Here, we stress that, when possible, population delineations and size estimates should be incorporated when identifying areas that are important for migratory species (*23*, *39*) or subject to particular threats (*40*, *41*). Our results show how such analyses can quantify political responsibility and reveal unexpected linkages, providing opportunities for improving the conservation of migratory species. The high seas are a global commons, where there is a lack of effective legal processes for ensuring the conservation and sustainable use of biodiversity. We show that migratory seabirds across the world connect numerous countries and the high seas, thereby contributing to the connectivity of the global ocean. As human endeavor in the marine environment expands, it becomes more important than ever that our systems of management and protection reflect this interconnected reality.

## MATERIALS AND METHODS

### Study taxa

For the purposes of this study, the term “albatrosses and large petrels” refers to all species in the albatross family Diomedeidae, and the genera *Macronectes*, *Ardenna*, *Calonectris*, and *Procellaria* of the family Procellariidae. This grouping was selected for the following reasons: 21 of these 40 species are listed as vulnerable, endangered, or critically endangered under the International Union for Conservation of Nature (IUCN) Red List Criteria and an additional 11 as near threatened (*42*); 29 are listed by ACAP (*10*), a high proportion face at-sea threats, namely incidental mortality in association with fishing, as well as direct competition with fisheries; last, these species are highly mobile and are relatively well studied across their ranges, and hence, the necessary tracking and population data are available for a global synthesis (*10*, *24*, *25*).

### Data assembly

Tracking data were obtained from the BirdLife International Seabird Tracking Database (www.seabirdtracking.org) and directly from collaborators. Data were sought from all the breeding aggregations for each species, with the goal of maximizing coverage of the global breeding population (Table 1 and data S3). Each “breeding population” was defined according to criteria adopted by ACAP as individuals of each species found breeding at each “island group”; the latter correspond with major archipelagos and reflects current biogeographic and political separation (*10*). For each breeding population with tracking data, we maximized coverage of the annual cycle (Table 1) by using data from light-level geolocation (Global Location Sensor or GLS), Platform Terminal Transmitters (PTTs), and Global Positioning System (GPS) devices. We obtained island group and global breeding population size estimates from the ACAP database (https://data.acap.aq/) and from the literature (data S4). We assembled requisite data for 39 of the 40 species of albatrosses and large petrels, with only the southern royal albatross *Diomedea epomophora* lacking the necessary data for analysis.

### Filtering and standardization

Because time intervals between locations differed among device types and datasets, we used a single location per day (i.e., the lowest common interval) from each individual track, nearest to local noon, for analysis. GLS, PTT, and GPS devices provide positions in different ways, resulting in different levels of spatial accuracy. Data from GLS devices are used to estimate location based on day length and the relative timing of local noon and have the highest spatial error, of around SD ±186 km (*43*). We applied a series of filters to GLS datasets in addition to those used by data contributors. We also performed a resampling procedure to test the sensitivity of our measures of the importance of political areas to spatial error and concluded that the effects on estimated richness and time spent were very small and had no substantial effects on the results (see Supplementary Materials and Methods for details and fig. S3 for results). Nine species (all *Diomedea* and *Phoebetria* spp. and gray-headed albatross *Thalassarche chrysostoma*) breed biennially, and because tracking data coverage during the nonbreeding year is generally poor, we retained only data from the first 365 days following logger attachment for each individual track. Hence, all results pertain to the distribution of adult birds during a breeding year.

After filtering, there were a total of 842,527 tracking days available for analysis from 5775 individual birds (Fig. 2A, fig. S1, and data S4). These data represent movements sampled between 1989 and 2017, across 87 breeding populations from 39 of the 40 species of albatrosses and large petrels (Table 1 and data S3). These populations represent a total of 44 million of the estimated 58 million breeding albatrosses and large petrels worldwide and a mean of 85% (range, 3 to 100%) of the global breeding population per species (Table 1). We identified the national jurisdictions and high seas areas visited by each breeding population for each month of the year (see the “Analysis” section); we considered months with fewer than 10 unique tracking days to be unrepresentative and removed them from the analysis. To test the sensitivity of this threshold, we reran the analysis with a more conservative threshold of at least 10 tracking days across five different individuals. This did not alter any major patterns or conclusions but excluded valuable data for several important populations from which few birds have been tracked; therefore, we opted to use the less conservative threshold (see data S5, A and B, and S6, A and B). Combined tracking samples for each population spanned a mean of 300 days (SD ±56) of the annual cycle (Table 1). After filtering unrepresentative months, tracking data allowed for the calculation of the national jurisdictions and high seas areas visited during a total of 94% of the annual cycle of 44 million albatrosses and large petrels and a mean of 76% (range, 3 to 100%) of the annual cycle per species (Table 1). Therefore, our results pertain to 41.3 million of a possible total of 58 million (71%) bird-years.

### Analysis

Each daily location estimate was assigned to a maritime zone (herein used when simultaneously referring to the following areas: EEZs, the high seas as a whole, and the areas of legal competence of RFMOs in the high seas). First, this was done for national jurisdictions and the high seas as a whole, using a spatial union between country land borders and EEZs (VLIZ 2020, available at http://marineregions.org). We considered national jurisdictions to be the aggregated area of EEZs (and territorial waters) under a single country, including therein any overseas dependencies. Then, the analysis was run again, attributing points in the high seas to the areas of competence of the RFMOs, available at http://fao.org/geonetwork/ (fig. S2). Areas with overlapping territorial claims were left named with all claimant countries listed (e.g., “disputed – Russia/Japan”), unless they had breeding colonies with tracking data, which was only the case for the Falklands/Malvinas, South Georgia/Georgias del Sur, and the Chafarinas/Zafarin Islands. For these cases, the analysis was run twice with breeding populations alternately assigned to each disputing country (see alternate results in data S5, C and D, and S6, C and D).

#### Species richness

Species richness was calculated for each national jurisdiction and the high seas as a whole based on tracking data occurrence and reflects the number of (i) breeding species, (ii) visiting species, and (iii) species that both breed locally and visit from elsewhere. To visualize this pattern in space, maps were constructed by binning total richness on a global hexagonal grid of with cell-center spacing of 495 km (SD ±30) which is a grid resolution large enough to encompass the average error of GLS devices (*43*). Calculations of breeding species richness for each country reflect only the breeding populations with available tracking data to ensure a valid comparison with the number of visiting species; therefore, this will be an underestimate of true breeding richness in certain cases. Using information on all known breeding countries for each species, we calculated the degree of underestimation in our calculations of breeding richness per country.

#### Time spent estimation

To reflect the relative abundances of albatrosses and large petrels in each maritime zone, we estimated the amount of time spent by each breeding population in a year. Time spent was first calculated for each month to account for uneven sample sizes and shifting seasonal distributions and then summed and expressed in “bird-years.” Each daily location within a maritime zone was considered one bird-day spent therein. The total amount of time spent (*T_spme_*; in bird-months) by the breeding population *p* of species *s* in maritime zone *e* was calculated as

$$T_{\text{spme}}=\frac{\sum_{i=1}^{i_{\text{max}}}(\frac{D_{\mathrm{ime}}}{D_{\mathrm{im}}})\;}{n_{m}}*N_{p}$$ (1)

where *D_ime_* represents the number of days an individual *i* spent in maritime zone *e* in month *m*, and *D_im_* represents the total number of days individual *i* was tracked in month *m*. This proportion of days spent in each maritime zone was then averaged across all tracked individuals (*i*_1_ to *i*_max_) by dividing the sum of proportions by the number of tracked individuals for a species and population *n_m_* in month *m*. The average proportion of monthly time spent per tracked individual was then multiplied by the breeding population size *N_p_* to extrapolate to the total amount of time (in bird-months) that breeding population *p* of species *s* spends in maritime zone *e* in month *m*.

For example, an individual (*i*) Cory’s shearwater *Calonectris borealis* (*s*) from the breeding population in Madeira (*p*) spent 10 of a total of 31 tracking days (32% of the time) in the high seas $\left(\frac{D_{\mathrm{ime}}}{D_{\mathrm{im}}}\right)$during January (*m*). In this way, we calculated the proportion of days spent in the high seas for each of the 72 tracked individuals (*n_m_*) of the same species and population. Next, we calculated the mean proportion of time spent across these 72 individuals, estimating that these birds spent, on average, 17% of January in the high seas. Multiplying this mean proportion by the breeding population size of 66,080 individuals (*N_p_*) results in a monthly total of 11,284 bird-months spent in the high seas in January (*T_spme_*).

#### Annual time spent

We then summarized the monthly time spent across all months to estimate the standalone importance of maritime zones *e* in terms of the total amount of time spent in a year (*T_e_*; in bird-years) by the global community of albatrosses and large petrels

$$T_{e}=\sum_{p=1}^{p_{\text{max}}}\frac{\sum_{m=1}^{m_{\text{max}}}(T_{\text{spme}})}{12}$$ (2)

where the total time spent within each month (*T_spme_*) was summed across the months for which tracking data were available (*m*_max_) and divided by 12 to convert the unit from bird-months to bird-years. The number of bird-years per population was then summed across all populations in maritime zone *e* to give an estimate of the total annual time spent there by all species.

Continuing our example of Cory’s shearwaters from Madeira, the total monthly time spent in the high seas (*T_spme_*) was calculated for the 11 months of the year where tracking data existed (*m*_1_ *to m*_max_) and then divided by the full 12 months of the year to convert the unit from bird-months to bird-years. To put this species- and population-specific pattern in the global context, we added the resulting total amount of time spent by Cory’s shearwaters to the values of all other breeding populations visiting the high seas (*p*_1_ *to p*_max_), giving the total amount of time spent in the high seas in a year by the global community of albatrosses and large petrels (*T_e_*).

Since tracking data were unavailable for some months of the year for certain populations, *T_e_* was underestimated in these cases because the unit conversion still divided the sum of all tracking months by 12 rather than the number of months for which tracking data were available; however, we preferred this conservative approach because calculating over the full year avoided extrapolating occurrence patterns into unsampled periods. The sum of *T_e_* across all maritime zones therefore equals the population size of all breeding populations with tracking data minus the untracked portions of the year for each population (i.e., 41.3 million bird-years estimated for 44 million birds; see the “Filtering and standardization” section). To visualize the time spent pattern in space, we aggregated the annual time spent over a hexagonal grid with cell-center spacing of 495 km (SD ±30) using the same equation (i.e., where *e* signified grid cells).

#### Network analysis

To estimate the strength of connection between breeding-origin countries and other visited maritime zones, we developed an index based on the percentage of annual time spent per maritime zone by populations of different breeding origins. The proportion of annual time spent *G_soe_* in maritime zone *e* by species *s* breeding under the jurisdiction of origin country *o* was calculated as

$$G_{\mathrm{soe}}=\frac{\sum_{p=1}^{x}\sum_{m=1}^{m_{\text{max}}}(T_{\text{spme}})}{N_{s}*12}$$ (3)

where the total within-month time spent (*T_spme_*) was summed across all months and all tracked populations *x* of a given species breeding in country *o* and converted from bird-months to bird-years by dividing by 12. This annual time spent value was then divided by the global population size *N_s_* of that species to calculate the proportion of annual time spent in each maritime zone by all individuals of said species.

In our example of Cory’s shearwaters from Madeira, we first added the total amount of time spent in the high seas in each month (*T_spme_*) across all tracking months of the year, resulting in 115,844 bird-months. Similarly, we calculated 1,988,600 bird-months spent in the high seas across the year for Cory’s shearwaters from the Azores and 4771 bird-months for the Berlengas population, which resulted in a total of 2,109,215 bird-months or 175,768 bird-years for all breeding populations of Cory’s shearwater falling under the jurisdiction of Portugal. We then divided the latter value by the global population size of 423,672 individuals of that species to estimate that the proportion of time spent (*G_soe_*) in the high seas (*e*) attributable to Cory’s shearwaters (*s*) of Portuguese breeding origin (*o*) was 41.5% of the annual time for the species.

Then, the strength of connection between each breeding-origin country and visited maritime zones was calculated as the percentage of annual time spent in each zone summed across all the species breeding in each country

$$G_{\mathrm{oe}}=\sum_{s=1}^{s_{\text{max}}}G_{\mathrm{soe}}*100$$ (4)

where *G_oe_* indicates the strength of the connection between breeding-origin country *o* and visited maritime zone *e* with respect to the global breeding population for each species and to the breeding community in each country. The connections were ranked by strength (*G_oe_*), and the strongest links were then plotted in a network diagram. We represented the top connections per breeding-origin country in the network as (i) the top five links in the country-to-country and the high seas analysis and (ii) the top three links in the country-to-RFMO competence area analysis (see tables S6 and S8 for the full set of connections). A virtual application is available at https://birdlifeseabirds.shinyapps.io/seabird-connections/ to facilitate viewing the specific connections albatrosses and large petrels create between different national jurisdictions and high seas areas, including RFMOs. All analyses were run using the R statistical computing environment; maps were made using the R packages “dggridR,” “ggplot2,” and “sf,” and networks were constructed using “ggraph” (*44*–*48*). Analysis scripts are available at https://github.com/MartinBeal/political_connectivity.

## SUPPLEMENTARY MATERIALS

Supplementary material for this article is available at http://advances.sciencemag.org/cgi/content/full/7/10/eabd7225/DC1
